# Assessment of prevalence and distribution of congenital missing teeth among patients visiting tertiary care hospital: A radiographic study

**DOI:** 10.1016/j.jobcr.2024.04.005

**Published:** 2024-05-04

**Authors:** Khushboo Arif, Vinay Kumar Gupta, Gaurav Mishra, Sumit Kumar, Atrey Pai Khot, Sonal Bhatia, Ranjit kumar Patil, Abhishek Singh, Mohammad Imran Khan

**Affiliations:** a Department of Public Health Dentistry, Faculty of Dental Sciences, King George's Medical University, U.P, Lucknow, India; b Department of Oral Medicine & Radiology, King George's Medical University, U.P, Lucknow, India; c Department of Community Medicine & Public Health, King George's Medical University, U.P, Lucknow, India; d Department Of Oral & Maxillofacial Pathology, Career Post Graduate Institute Of Dental Sciences & Hospital, Ghailla, Lucknow, India

**Keywords:** Hypodontia, Panorami cradiography, Toothagenesis

## Abstract

**Introduction:**

Dental Agenesis is the usual developmental dental anomaly involving both primary and permanent dentition but most commonly it affects the permanent teeth. Genetic mutations in genes like MSX, PAX9,TGFA and AXIN2 are the likely primary contributors to tooth agenesis. Identifying the prevalence and distribution of congenital missing teeth allows for early detection and intervention which is crucial for preventing or mitigating potential dental issues that may arise due to missing teeth.

**Aim & objectives:**

To assess the prevalence and distribution of congenitally missing teeth across different quadrants of the jaw among patients visiting to the Dental out patient department at Tertiary Care Centre of Lucknow city.

**Material & method:**

A Questionnaire and orthopantomogram based cross-sectional study was conducted on both male and female patients aged between 15 and 30 years, coming for evaluation of their dental health problems to the hospital. Written informed consent was obtained.

**Results:**

The overall prevalence of CMT was found to be 33.3 %. The significant difference was observed in proportion of CMT between Maxilla and Mandible sites (p = 0.008). Higher prevalence was in females compared to males for both maxillary and mandibular congenital missing teeth. (p = 0.020).

**Conclusion:**

The most common CMT were third molars followed by lateral incisors. The insights derived from the study would aid dental professional in gaining a deeper comprehension of tooth agenesis.

## Graphical abstract

**Figure:**
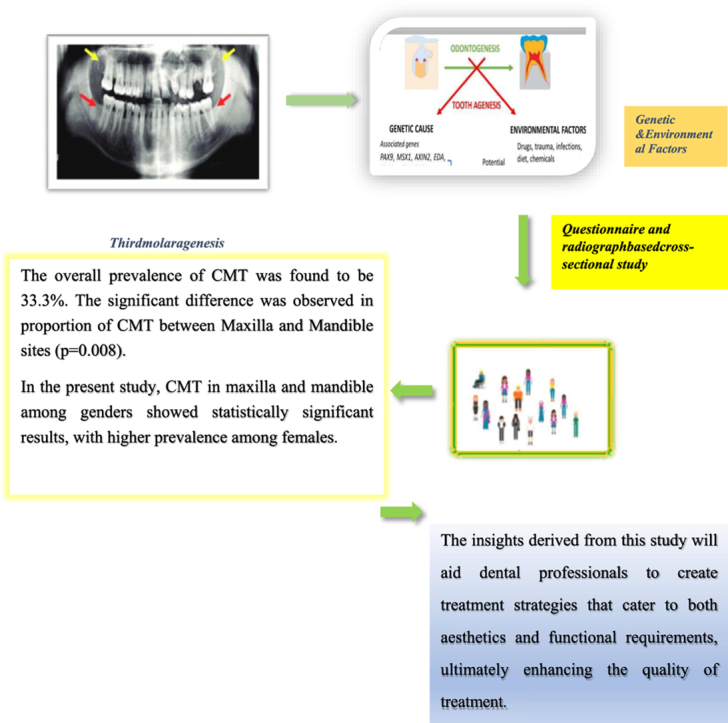

## 1 Introduction

The well-being of oral health significantly impacts the overall realm of public health.^1^ Tooth agenesis, Congenital Dental Aplasia or Dental Agenesis is the usual developmental dental anomaly involving both primary and permanent dentition but most commonly it affects the permanent teeth. This phenomenon, occurring either in isolation or as part of a syndrome, represents a prominent irregularity in dental development.^2^ Dental agenesis is a condition characterized by the absence of certain teeth, referred to as "missing teeth."Anodontia, on the other hand, specifically signifies the complete absence of all teeth, whereas hypodontia is characterized by the congenital absence of a range of 1–6 teeth. It can manifest either within the context of a recognizable genetic syndrome or as an independent, non-syndromic trait.^3^ Oligodontia pertains to the absence of over six teeth, encompassing those that have not emerged in the oral cavity or show any indication of development on clinical and radiographic assessments.

Congenital missing teeth (CMT) are generally not regarded as a significant health concern. The intricate interplay among genetic, epigenetic, and environmental elements during dental development can lead to disruptions in odontogenesis.^4^ The potential consequences encompass challenges in speech articulation, unfavourable aesthetics, periodontal trauma, and inadequate jaw bone growth. Disruptions in the Dental lamina during embryonic development, compression during germ formation, environmental influences, hormonal fluctuations, radiotherapy chemotherapy maternal medications and nutritional imbalances in infancy, may contribute to CMT. Dental agenesis is also observed in conditions like Down syndrome, Reiger syndrome, hypohidrotic ectodermal dysplasia, and cleft lips.

Genetic mutations in genes like MSX, PAX 9, TGFA (associated with cell proliferation, differentiation, and development) and AXIN2 are the likely primary contributors to tooth agenesis.^1^ Situated on chromosome 14, PAX9 (Paired box) serves as a regulatory element in dental development, and mutations in this gene are linked to instances of tooth agenesis.^5^

An optimal CMT diagnosis requires a radiograph, clinical and dental cast examination. Through radiographic analysis, the absence of dental buds becomes evident, as they are not discernible in the radiograph images.^6^ Identifying the prevalence and distribution of congenital missing teeth allows for early detection and intervention which is crucial for preventing or mitigating potential dental issues that may arise due to missing teeth. It offers valuable insights into genetic predispositions, environmental factors, and potential connections to other health conditions. Guided by this research, public health initiatives will be aimed at promoting oral care leading to more targeted and effective strategies for enhancing dental well-being. Therefore, prompt diagnosis could help in developing an efficient treatment plan and averting more complex issues. Additionally, it contributes to dental research, education, and the development of clinical guidelines. Public health implications include raising awareness and improving access to dental care. Ultimately, this research aims to enhance oral health outcomes, prevent complications, and guide informed decision-making for affected individuals and their families.

## 2 Aim and objectives

To assess the prevalence and distribution across different quadrants of the jaw of congenitally missing teeth, and its associated dental anomalies among patients visiting the Dental outpatient department at Tertiary Care Centre of Lucknow city.

## 3 Material and method

This current research comprised a hospital-based, cross-sectional descriptive study that relied on the questionnaire and ortho pantomo graph radiograph to evaluate the prevalence and distribution of congenitally missing teeth among individuals who had visited the Out Patient Department of the Faculty of Dental Sciences in Lucknow Uttar Pradesh, India for assessment of their dental problems through ortho panto mogram (OPG).

Patients both male and female aged between 15 and 30 years, who gave consent and had come to the hospital for evaluation of their dental health problems and who were advised for ortho pantomography constituted the population of the study.

On the basis of the study^7^ advocating the significant prevalence of congenital missing teeth (10.37 %) and factors associated with it among patients attending dentistry centres in Baghdad. Therefore, based on the sample size calculation the minimum representative sample size required was 138. The final sample size was increased to 243 for improving the external validity of the study. A written informed consent was obtained from each participant after a comprehensive explanation of the methodology prior to enrolment in the study. The eligible and willing participants radiographs were assessed including the third molars. Patients with previous traumatic dental avulsion, extracted teeth, periodontal problems and undergoing orthodontic treatment were excluded from the study. Absolute confidentiality and anonymity were assured to the patients & ethical approval was obtained from the institutional ethical committee.

### 3.1 Data collection

Eligible and interested patients were asked questions related to their demographic details. This tool was designed to evaluate the occurrence of congenitally absent teeth in patients recommended for orthopantomogram imaging. The criterion for the study is formulated to make the study, simple and clear. Questionnaires were administered by the investigator to the participants. The study focuses on individuals seeking dental health evaluation at the dental hospital for addressing their oral health concerns.

The questionnaire consists of three parts:

**First part**: It consists of socio-demographic details. **Second Part:** Comprises of medical and dental history **Third Part**: It constitutes the radiographic details.

### 3.2 Assessment of proforma

Patients had undergone assessment based on congenitally missing teeth, taking into account factors such as age, gender and the arrangement pattern of their missing teeth. Radiographs were reviewed using an X-ray viewer and evaluated by the Principal investigator. A tooth was considered congenitally missing if its O.P.G. (orthopantomograph) radiograph showed no calcification in the corresponding region.

### 3.3 Statistical analysis

Analysis was performed on SPSS software (windows version 24.0). The relation between CMT with age, gender and jaws was analyzed using Pearson's chi-square test. The level of significance will be set at 0.05.

## 4 Results

### 4.1 Age distribution

• **15–20 years**: There are 73 participants in the age group of 15–20 years, which accounts for 30.0 % of the total study population. This group represents young adults in the early stages of their reproductive years.
• **21**–**25years**: The age group of 21–25 years comprises 74 participants, making up 30.5 % of the total participants. This group represents a similar age range to the previous group and is also within the early reproductive years.
• **26–30 years**: The largest age category consists of 96 participants in the age range of 26–30 years, representing 39.5 % of the total study population. This group includes participants in their late twenties to early thirties.

### 4.2 Gender distribution

**Female**: The study includes 119 female participants, constituting 49.0 % of the total study population. This group predominantly comprises women, reflecting nearly half of the study participants.

**Male:** There are 124 male participants, making up 51.0 % of the total participants. This group consists of men, representing just over half of the study's gender distribution.

This detailed breakdown of the data provides a comprehensive view of the age and gender demographics within the study, offering a clearer understanding of the characteristics of the participant population.

This data offers valuable insights into the distribution of various dental conditions and issues across different genders. The study population was divided into two categories: Female and Male.

**Partially Edentulous**: The data demonstrates variations in the prevalence of partial edentulism between females and males. For the anterior condition, 21.0 % of females and 4.0 % of males were affected, while for both anterior and posterior, 3.4 % of females and 1.6 % of males had this condition. A majority of both females (56.3 %) and males (75.0 %) had no partial edentulous condition. The chi-square test indicates a significant difference in partial edentulism between genders (chi-square = 18.15, p < 0.001), with a higher prevalence among females.

**Number of Missing Teeth**: The distribution of missing teeth, including none, one, two, and three or more than three, varied between females and males. Notably, 56.3 % of females and 75.0 % of males had no missing teeth. Additionally, 17.6 % of females and 8.9 % of males had one missing tooth, 10.1 % of females and 8.1 % of males had three or more than three missing teeth and 16.0 % of females and 8.1 % of males had two missing teeth. The chi-square analysis indicates a significant difference in the number of missing teeth between genders (chi-square = 10.23, p = 0.017), with a higher prevalence of missing teeth among males.

**Pattern of Missing Teeth:** The distribution of patterns of missing teeth, such as bilateral, both, unilateral left side, unilateral right side, or none, showed variations between females and males. The chi-square test revealed a significant difference in the pattern of missing teeth between genders (chi-square = 14.43, p = 0.006), with a higher prevalence of certain patterns among males.

**Location of Missing Teeth:** The data indicates differences in the location of missing teeth between females and males. Specifically, 11.8 % of females and 8.1 % of males had missing teeth in both locations, 9.2 % of females and 4.0 % of males had missing teeth in the mandible, and 22.7 % of females and 12.9 % of males had missing teeth in the maxilla. The majority of both females and males had no missing teeth. The chi-square analysis revealed a significant difference in the location of missing teeth between genders (chi-square = 9.86, p = 0.020), with variations in the prevalence of missing teeth in different locations between the two genders.

The data regarding congenital missing teeth (CMT) highlights the prevalence of this condition in the study population. The overall prevalence of CMT was found to be 33.3 %. Further, the statistics reveal that among the participants, 65 individuals, representing 26.7 % of the total, had congenital missing teeth (CMT) in the maxilla. Additionally, 34 participants, constituting 14.0 % of the total, had congenital missing teeth in the mandible. A significant difference was observed in the proportion of CMT between Maxilla and Mandible sites (p = 0.008).

The data presents the prevalence of congenital missing teeth in both the axilla and mandible across two gender groups, females and males. It is noteworthy that specific tooth locations with congenital missing teeth are also detailed within each group.

### 4.3 Congenital missing teeth in the Maxilla

In the female group, 67.2 % of individuals had no congenital missing teeth in the maxilla. Within this group, some instances of congenital missing teeth were observed in specific tooth locations, such as 12, 12; 14, 12; 22, 13, 13; 23; 28, 18, 18; 24; 28, 18; 28, 18; 28, 18, 22, and 28.

For the male group, 79.0 % had no congenital missing teeth in the maxilla. Specific tooth locations with congenital missing teeth in this group included12,12; 22,18,18; 28, and 28.

The chi-square analysis for congenital missing teeth in the maxilla showed no statistically significant difference (chi-square = 18.45, p = 0.103) between the genders.

### 4.4 Congenital missing teeth in the mandible

Among females, 80.7 % had no congenital missing teeth in the mandible. In this group, some instances of congenital missing teeth were found in tooth locations 32, 38, 42, 43, 44, and 48; 38. In the male group, 91.1 % had no congenital missing teeth in the mandible. Instances of congenital missing teeth were noted in tooth locations 42 and 48; 38. The chi-square analysis for congenital missing teeth in the mandible demonstrated a statistically significant difference (chi-square = 15.06, p = 0.020) between female and male genders.

In summary, the data showcases the distribution of congenital missing teeth in the maxilla and mandible among female and male individuals, with some variations in specific tooth locations, particularly in the mandible, where a significant difference was observed between the two genders.

This data provides insights into the distribution of congenital missing teeth in both the axilla and mandible among different age groups which are categorized into three age ranges: 15–20 years, 21–25 years, and 26–30 years and among genders, which are categorized as female and male. The chi-square analysis indicates no significant difference in the prevalence of congenital missing teeth in the maxilla and mandible among different age groups (chi-square = 4.30, p = 0.116) & (chi-square = 2.35, p = 0.309) respectively.

**Congenital Missing Teeth in the Maxilla**: The data shows the prevalence of congenital missing teeth in the maxilla among different genders. Among females, 32.8 % had congenital missing teeth in the maxilla, while 67.2 % did not. In contrast, among males, 21.0 % had congenital missing teeth in the maxilla and 79.0 % did not have this condition. The chi-square analysis indicates a statistically significant difference in the prevalence of congenital missing teeth in the maxilla between females and males (chi-square = 4.32, p = 0.038), with a higher prevalence in females.

**Congenital Missing Teeth in the Mandible**: The data also presents the prevalence of congenital missing teeth in the mandible across different genders. Among females, 19.3 % had congenital missing teeth in the mandible, while 80.7 % did not. For males, 8.9 % had congenital missing teeth in the mandible, and 91.1 % did not have this condition. The chi-square analysis suggests a statistically significant difference in the prevalence of congenital missing teeth in the mandible between females and males (chi-square = 5.52, p = 0.019), with a higher prevalence in females.

The data provides valuable information about the occurrence of congenital missing teeth in both the maxilla and mandible based on gender, indicating a higher prevalence of this condition in females compared to males for both maxillary and mandibular congenital missing teeth.

## 5 Discussion

Congenital missing teeth (CMT) are the most widespread dental anomaly carrying the potential for cosmetic and functional challenges that may require a complex and expensive multidisciplinary treatment approach.^8^ This study was conducted on 243 participants consisting of 119 females and 124 males aged between 15 and 30 years who visited the outpatient department of the Faculty of Dental Sciences, for assessment of their dental problems through orthopantomogram (O.P.G).

**Fig. 1:**
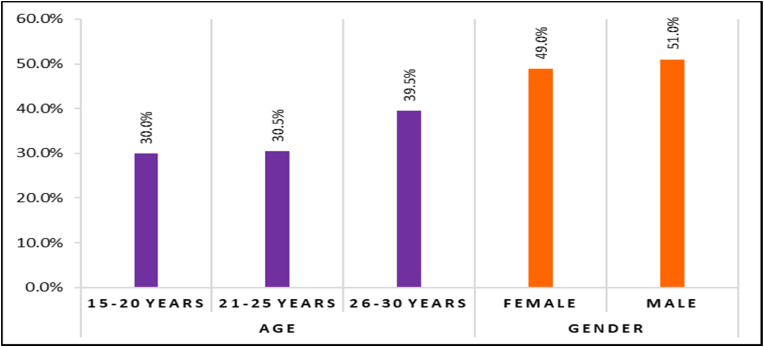
Distribution of Cases according to age and gender.

Age of the study participants ranged from 15 to 30 years, mostly age group 25–30 (39.5 %) followed by 21–25 years (30.5 %) and 15–20 years (30.0 %) (Fig. 1). The prevalence of CMT was highest in 15–20 years (35.6 % in maxilla and 19.2 % mandible). The upper age limit chosen was 30 years, to mitigate the risk of false positive results stemming from factors such as extractions, tooth loss, and other issues that could be erroneously associated with a genesis. The findings of the present study indicate that there are no substantial age-related differences in the occurrence of CMT across various age categories (chi-square = 4.30 and 2.35, p = 0.116 and 0.309 in the maxilla and mandible, respectively).(Table 3) The possible reason behind this is that this condition is typically determined during the early stages of tooth development. As the genetic factors are established before birth, and once the number and type of teeth are determined they remain relatively constant throughout life. Therefore, variations in the occurrence of a condition among individuals are primarily influenced by genetic and developmental variables rather than changes that occur with age.

**Table 1:** Association of gender with teeth problems.

Variable		Gender			
		Female		Male	
		No.	%	No.	%
	Anterior	25	21.00 %	5	4.00 %
	Both anterior & posterior	4	3.40 %	2	1.60 %
	None	67	56.30 %	93	75.00 %
Partially Edentulous	Posterior	23	1.30 %	24	19.40 %
	significance	chi sq = 18.15, **p<0.001**			
	None	67	56.30 %	93	75.00 %
	One	21	17.60 %	11	8.90 %
					
	Three or more than three	12	10.10 %	10	8.10 %
No. of Missing Teeth	Two	19	16.00 %	10	8.10 %
	significance	chi sq = 10.23, **p=0.017**			
	Bilateral	17	14.30 %	12	9.70 %
	Both	8	6.70 %	8	6.50 %
	None	65	54.60 %	93	75.00 %
	Unilateral Left Side	15	12.60 %	4	3.20 %
Pattern of Missing teeth	Unilateral Right Side	14	11.80 %	7	5.60 %
	significance	chi sq = 14.43, **p=0.006**			
	Both	14	11.80 %	10	8.10 %
	Mandible	11	9.20 %	5	4.00 %
Location of Missing Teeth	Maxilla	27	22.70 %	16	12.90 %
	None	67	56.30 %	93	75.00 %
	significance	chi sq = 9.86, **p=0.020**

**Table 2:** Distribution of Cases according to CMT Type and its association with Gender.

Variable		Gender						
					Female		Male	
Congenital missing teeth		No.	%		No	%	No	%
					80	67.20 %	98	79.00 %
	2	17	7.00 %	12	4	3.40 %	1	0.80 %
	13	4	1.60 %	12; 14	2	1.70 %	0	0.00 %
	14	2	0.80 %	12; 22	6	5.00 %	4	3.20 %
Maxilla	18	31	12.80 %	13	2	1.70 %	0	0.00 %
	212	12	4.90 %	13; 23; 28	2	1.70 %	0	0.00 %
	23	2	0.80 %	18	2	1.70 %	2	1.60 %
	24	2	0.80 %	18; 24; 28	2	1.70 %	0	0.00 %
	28	38	15.60 %	18; 28	12	10.10 %	9	7.30 %
				18; 28	0	0.00 %	2	1.60 %
				18; 38	0	0.00 %	2	1.60 %
				22	2	1.70 %	0	0.00 %
				28	5	4.20 %	6	4.80 %
	**Significance**			chi sq = 18.45, p = 0.103				
					96	80.70 %	113	91.10 %
	32	3	1.20 %	32	3	2.50 %	0	0.00 %
	38	21	8.60 %	38	5	4.20 %	0	0.00 %
	42	9	3.70 %	42	7	5.90 %	2	1.60 %
Mandible	43	1	0.40 %	43	1	0.80 %	0	0.00 %
	44	2	0.80 %	44	0	0.00 %	2	1.60 %
	48	16	6.60 %	48; 38	7	5.90 %	7	5.60 %
	**Significance**		chi sq = 15.06, p = 0.020

**Table 3:** Association of age & gender with CMT.

		Age						Gender			
Variable		15–20years		21–25years		26–30years		Female		Male	
		No.	%	No.	%	No.	%	No	%	No	%
Congenital missing teeth [Maxilla ]	No	47	64.40 %	58	78.40 %	73	76.00 %	80	67.20 %	98	79.00 %
	Yes	26	35.60 %	16	21.60 %	23	24.00 %	39	32.80 %	26	21.00 %
	significance	chi sq = 4.30, p = **0.116**						chi sq = 4.32, **p=0.038**			
Congenital missing teeth [Mandible]	No	59	80.80 %	65	87.80 %	85	88.50 %	96	80.70 %	113	91.10 %
	Yes	14	19.20 %	9	12.20 %	11	11.50 %	23	19.30 %	11	8.90 %
	significance	chi sq = 2.35, **p=0.309**						chi sq = 5.52, **p=0.019**			

**Fig. 2:**
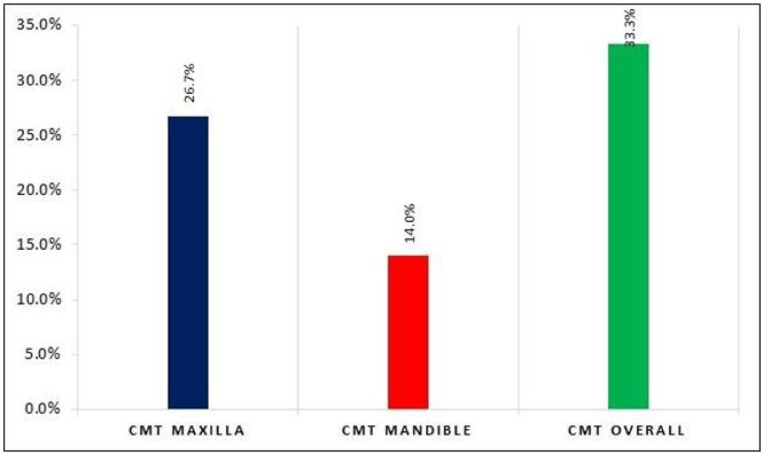
Prevalence of CMT CMT=Congenital missing teeth.

In the present study, the result showed the overall prevalence of CMT to be 33.3 % (Fig. 2). The prevalence of hypodontia was higher because we have included the third molars as compared to the studies reported by Altan H et al. (2019)^9^ and Musaed et al. (2019)^10^ where the prevalence of CMT were (14.1 %, and 16.3 %) respectively excluding the third molars. Nevertheless, the prevalence of CMT, including the third molars, was lower than what was reported in the research conducted by Scheiwiller M et al. (2020)^11^ and Sheikhi M et al. (2012)^12^, where they found rates of 50.8 % and 45.7 % respectively. The discrepancy in prevalence can be ascribed to differences in the demographic under study, genetic variables, sample size, and the inclusion or exclusion of radiographs and third molars during the examination procedure.

Out of 243 patients, there were (49 %) females and (51.0 %) males (Fig. 1). The prevalence of CMT in the present study was (32.8 % & 19.3 %) in females and (21.0 % & 8.9 %) among males in the maxillary and mandibular arches respectively. In the present study, CMT in maxilla and mandible among genders showed statistically significant results, with higher prevalence among females. This was in synchrony with the studies reported by Schonberger. S et al. (2023).^6^ Sisman Y (2007)^13^ and Kathariya et al. (2013)^14^ showed contrasting findings regarding the prevalence of hypodontia, with a higher occurrence observed in males compared to females. The higher prevalence reported in females may be linked to biological variances, such as a smaller jaw, which could potentially be altered by environmental variables. Additionally, females may have a greater need for orthodontic treatment due to their specific concern for their looks. Additionally, it can be ascribed to several causes such as genetic susceptibility, hormonal impacts, and variations in tooth growth.

The study revealed that the CMT was higher in the maxillary arch (26.7 %) compared to the mandibular arch (14.0 %) as shown in Fig. 1. Kapdan et al. (2012)^15^ and Vahid D et al. (2010)^16^ attained a higher prevalence of CMT in the maxillary arch. In contrast, Chung et al. (2008)^17^ and Hassan et al. (2014)^18^ found CMT in mandible more than maxilla. Schonberger et al. (2023)^6^ and Chib et al. (2022)^19^ found that the prevalence of CMT was higher, with 56.8 % in total, 55 % in the maxilla, and 43.2 % in the mandible. Maxillary teeth are more affected by genetic mutations, molecular mechanisms, and environmental variables (teratogens or development problems). This might be due to the fact that maxillary teeth form earlier in embryonic development and any disruptions in this process can lead to hypodontia.

The prevalence of CMT in this study is highest in the posterior (19.3 %), followed by the anterior (12.3 %) and in both (2.5 %). A chi-square test revealed no statistical difference for this condition among age groups. (p = 0.18). Similar results were reported by Uzuner et al. (2013)^20^ where they found CMT more in the posterior region (55.2 %). Opposite findings were reported by Amini F et al. (2012)^21^ where they showed higher prevalence in the anterior segment. A higher prevalence of partial edentulism was found more in females than males (Anterior = 21 %, 4 %; Posterior = 19.3 %, 19.4 %; both = 3.4 %, 1.6 %) with significant statistical difference between genders. This may beat tribute to the tooth development timing as anterior teeth develop before posterior teeth, any disturbance that affects this developmental process is more likely to impact the developing posterior teeth **(**Table 1).

Our findings reveal that the number of participants with single missing teeth (13.2 %), two (11.9 %), three or more (9.1 %). The distribution of one, two, three or more missing teeth was quite similar across the ages with no significant difference. There is a higher prevalence of number of missing teeth in males as the distribution of teeth among the genders is considered with significant difference. Aktan A (2010)^22^ reported similar results. In contrast, Galluccio et al. (2008)^23^ found that females had a higher likelihood of having more missing teeth. The variation in tooth development can be attributed to factors such as tooth type and position, as well as pre and postnatal influences and mutations in genes that regulate tooth growth.

In the present study, when the location of missing teeth is considered it was found to be more in the Maxillary arch (17.7 %), followed by both arches (9.9 %), and then in the mandible (6.6 %). Alike to this Uzuner et al. (2013)^20^ reported a higher rate of missing teeth in the maxilla (51.8 %) than in the mandible (45.9 %). Soni H K et al. (2018)^24^ reported mandibular hypodontia to be higher. The statistical analysis suggests that the prevalence of missing teeth and location was relatively consistent regardless of age. Females have a higher prevalence of missing teeth in Maxilla (22.7 %) and mandible (9.2 %) respectively in the current study. Similar findings were reported by Ali S et al. (2019).^25^ This is due to the fact that genetic variations are more common in females with maxillary dental arches are smaller than males and smaller arches do not have enough space for all teeth to develop properly leading to a greater prevalence of CMT in the maxilla.

Results in the current study, with Bilateral Agenesis (11.9 %), unilateral right side (8.6 %), unilateral left side (7.8 %), 6.6 % in both, revealed no statistical difference in the pattern of missing teeth across the age groups (p = 0.136) but observed higher prevalence of among males with statistical significance (p = 0.006) when pattern of missing teeth is considered. In this study, the prevalence of CMT in the right side of the arch was found to be more frequent than in the left side. The findings were in agreement with Uzuner et al. (2013)^20^ where they determined a higher prevalence (51.8 %) on the right side and 48.2 % on the left side. Rakshan V et al. (2014)^26^ detected no significant difference as the number of missing teeth in right and left sides were considered.

The most common bilateral agenesis in the present study were the maxillary third molars followed by mandibular third molars and maxillary lateral incisors. Kirzioglu et al. (2005)^27^ observed a higher prevalence of bilaterally missing teeth (73.2 %). It was noticed that missing third molars and incisors were significantly more frequent in females. In the current study, the least missing teeth were the second mandibular premolars and maxillary central incisors. It was recommended from the results of the study that unilateral agenesis was more common in the upper and lower lateral incisors whereas bilateral missing teeth were more common in maxillary third molars. Scheiwiller M et al. (2020)^11^ and Sheikhi M et al. (2012)^12^ reported similar results (50.8 % and 34.8 %). This implies that genetic factors influencing tooth agenesis may render the third molars more susceptible compared to other teeth. A diminished dentition may consequently be linked to nature's adaptation to accommodate the shortening of the dental arches.

The current study revealed that (28.4 %) maxillary, (15.2 %) mandibular third molars are missing more followed by lateral incisors (16.8 %) and first premolars (2.4 %) (Table 2). Contrasting results were reported by Ali S et al. (2019)^25^, where it showed that mandibular (70.34 %) followed by maxillary (63.42 %) third molars are missing. A higher prevalence of third molar agenes was reported by (50.8 %) by Schweiller et al. (2020).^11^ The CMT distribution between the genders was illustrated in Table 2 which depicted that wisdom tooth has the highest percentage followed by lateral incisors and first premolar with significant differences between genders in both the arches (p = 0.020). This certainty was because third molars are the last tooth to develop in the dentition and hence they show more agenesis and vulnerability to genetic and environmental factors during development.

Certain anomalies co-existed along with tooth agenesis,1.2 % reported supernumerary teeth. The results were in synchrony with Ziad et al. (2019).^10^ The presence of it might lead to developmental issues that may result in functional and esthetical concerns. Only 0.8 % of individuals were depicted to have concrescence teeth.

The prevalence of rotated teeth (2.5 %) was the highest among any other dental anomaly in the coeval study. This was in agreement with Bakhurji M et al. (2021).^2^ This information would be valuable for clinicians when assessing patients, enabling them to identify this common issue and be better equipped to provide effective management. Raising awareness, promoting early detection and providing accessible dental care are essential for improving the overall health of individuals and preventing potential complications.

The findings of this study contribute to the existing body of knowledge on tooth agenesis and provide a basis for further research in this field. Continued research into the genetic and environmental factors influencing CMT prevalence and distribution may lead to advancements in preventive strategies to improve patient care, diagnostic tools, and treatment modalities.

Limitations of the study include potential selection bias due to the small sample size and age range (15–30 years), reliance on self-reported data and orthopantomograms, lack of consideration for genetic and confounding factors, cross-sectional design of the study which may impact the study's generalizability and validity.

## 6 Conclusion

The prevalence of CMT was 33.3 %, there was a significant difference in the prevalence among genders. Agenesis occurred more bilaterally than unilaterally. Maxillary third molars emerged as the most frequently congenital missing teeth, followed by mandibular third molars and maxillary lateral incisors.

The insights derived from this study will aid dental professionals in gaining a deeper comprehension of tooth agenesis. This understanding would empower them to create treatment strategies that cater to both aesthetics and functional requirements, ultimately enhancing the quality of treatment.

## Sources of funding

Nil.
